# Lysenko and the Screwworm Fly—When Politics Interferes with Science and Public Health

**DOI:** 10.3390/ijerph17186687

**Published:** 2020-09-14

**Authors:** Carlos Brisola Marcondes, Angelo Canale, Giovanni Benelli

**Affiliations:** 1 Departamento de Microbiologia, Imunologia e Parasitologia, Centro de Ciências Biológicas, Universidade Federal de Santa Catarina, Florianópolis 88040-900, Brazil; cbrisolamarcondes@gmail.com; 2 Department of Agriculture, Food and Environment, University of Pisa, via del Borghetto 80, 56124 Pisa, Italy; angelo.canale@unipi.it

**Keywords:** blowflies, Calliphoridae, *Cochliomyia hominivorax*, eradication, genetic control, myiasis, sterile insect technique (SIT)

## Abstract

In the One Health scenario, a deep understanding of the dynamics potentially threatening the development and implementation of useful pest and vector management tools is of key importance. The New World screwworm fly, *Cochliomyia hominivorax* (Coquerel) (Diptera: Calliphoridae), is characterized by a wide host range. It acts as an important agent of myiasis in humans and warm-blooded animals in the Neotropics, and has been eliminated from a wide region through genetic methods. Of note, Serebrovsky had already proposed in 1940 the principles of autocidal control by the translocation of segments between two chromosomes, but his work was negated by Lysenko, based on the negation of Mendelian genetics. This entomological case study emphasizes the danger of politics interfering with science, a still contemporary hot issue. The negation of global warming or current pandemics are further examples of this noxious influence.

## 1. On Politics and Science: An Entomological Case Study

The One Health approach strongly supports interdisciplinary cooperation for designing and implementing programs, policies, legislation, and research in all facets of health care for humans, animals, and the environment [1,2,3,4]. The One Health approach has a highly promising potential for managing arthropod-borne diseases [5,6]. Within One Health, recognizing dynamics potentially threatening the development and implementation of useful pest and vector management tools is of key importance. More generally, the danger of politics and religion interfering with science represents a crucial historical issue. A wide number of evidences have been reported [7], including the ban of Galileo Galilei’s theories by the Roman Inquisition in 1615 [8], just to cite a major one (even if this should be contextualized in the unique circumstances of the Counter-Reformation Church, as stressed by some historians, see Wilson [9]).

However, one may argue: is this issue still contemporary? Unfortunately, the reply is in the affirmative, touching a number of issues of everyday life, as shown by the heavy impact of Brazilian politics on environmental policies [10], or—on a more local scale—by debated badger control practices adopted in Britain in the attempt to fight the spread of bovine tuberculosis [11]. Other examples relevant to everyday life include sex education and contraceptives not being accepted by religion [12], abortion and the debate about when life begins from a developmental biology perspective [13], issues associated with releasing genetically modified mosquitoes [14], as well as the use of insecticides for mosquito control during a disease outbreak (e.g., the ban of naled from several countries at the time of Zika virus epidemics [15]). Screening the literature about medical and veterinary entomology, an overlooked case study was found, which—to our mind—may represent a useful lesson for the future.

## 2. The New World Screwworm: A Successful Pest Management Story

**Figure 1:**
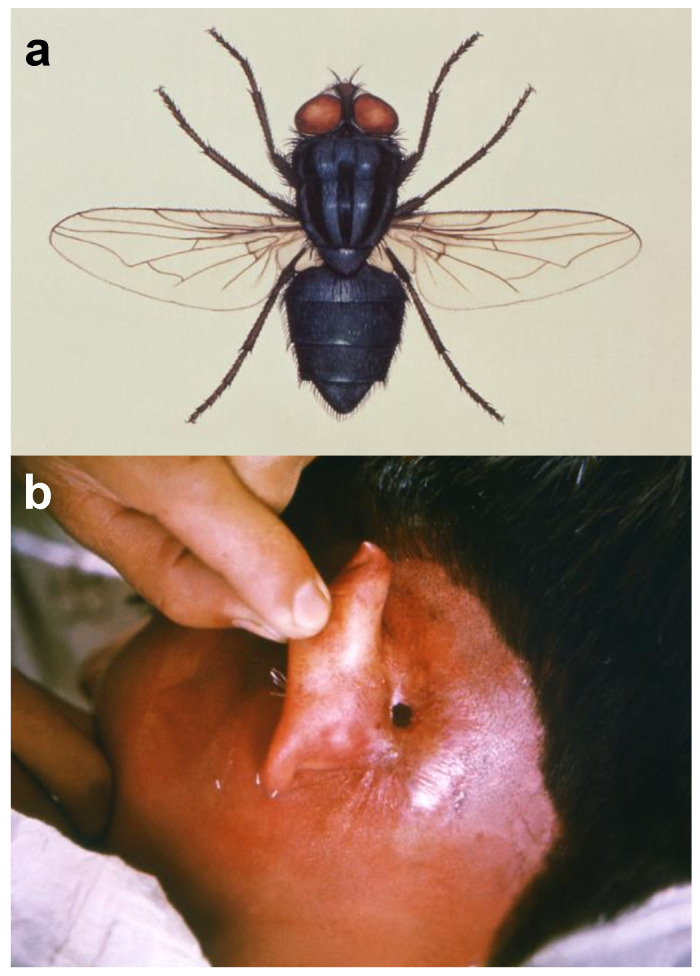
The New World screwworm, *Cochliomyia hominivorax* (Coquerel) (Diptera: Calliphoridae) (**a**); *C. hominivorax* can cause severe myases on humans, including head soft tissues burrowing post-oviposition inside the ear canal (**b**) (image credits, a: CDC-PHIL; b: Dr. K. Dewitt, CDC-PHIL).

The New World screwworm, also known as the American screwworm, *Cochliomyia hominivorax* (Coquerel) (Diptera: Calliphoridae) (Figure 1a), is characterized by an extremely wide host range; this species causes obligatory myiasis on humans (Figure 1b) as well as in animals of several species [16]. Notably, infestation by maggots of *C. hominivorax* represents a highly dangerous condition for companion animals [17] and livestock, with special reference to cattle in the Neotropics [18]. Therefore, the development of effective control actions to manage this pest has been of major economic importance [16].

Doramectin has been successfully employed for the treatment of *C. hominivorax* myiasis, while both the protection and residual action of ivermectin appeared to be shorter [19]. Other insecticides, including neonicotinoids, fipronil, spinosad, and the isoxazoline sarolaner, have been proposed to control this blowfly, mostly on companion animals [20,21,22,23]. However, resistance to organophosphorus and pyrethroid insecticides has been reported, and genes involved in such resistance have been studied [24,25,26].

On the other hand, baseline knowledge and real-world applications of biological control agents of *C. hominivorax* remain patchy. Biological autocidal techniques would be ecologically friendly, long lasting for the control, and more useful than the utilization of insecticides [27]. Due to autocidal control, the distribution of *C. hominivorax* is currently restricted to South America (except Chile) and some Caribbean islands [28]. Invasions were noticed and controlled in Libya [29] and Florida (USA) [30] utilizing the sterile insect technique (SIT). 

The autocidal control, through the liberation of sterilized males, has been very successful [31]. Male sterilization, coupled with other techniques (e.g., baiting of females), has led to *C. hominivorax* elimination from the Nearctic, reducing the distribution of this pest to the Neotropics [32]. The autocidal control of *C. hominivorax* has been adequately attributed to Edward F. Knipling, who worked on this subject since his graduation, in 1934 [33]. He and other researchers gradually improved the rearing of huge quantities of flies and methods of sterilization, and the fly was eradicated from all of North and Central America in the 1990s [30].

## 3. Earlier in the Soviet Union: Spradbery Facing Lysenko

However, as later highlighted by Spradbery [33], a Russian researcher, Alexander S. Serebrovsky, had already established the principles of autocidal control several decades before [34]. He proposed the utilization of chromosome translocation for autocidal control, but his theory and proposal, in the midst of the political turmoil of 1930s in the Union of Soviet Socialist Republics (USSR), were disregarded.

For many years, Trophim Lysenko (1898–1976), after some contribution to agricultural sciences in the 1920s, adopted an already known technique called vernalization (from Latin *vernus*, “of the spring”), which he called jarovization (яровизация in Russian, from “*jarovoi*”, meaning spring cereals), and became a highly influential biology researcher in the Soviet Union, in the midst of the scientific and political disputes in the 1930s, which caused spurges and condemnation of millions. He fought against Mendelian genetics, defended by his former chief Nikolai Vavílov, who was imprisoned and died in the Gulag in 1943 [35], and other geneticists. Stalin, an *ex machina* leader for scientific matters, liked his theories on Lamarckism, because they were the ideas of “a Soviet Man” [36]. In 1948, the discordance to environmentally acquired inheritance was formally prohibited [35]. After Stalin’s death in 1953, Lysenko’s influence was reduced until his retirement in 1965.

Although epigenetics has been sometimes shown as a resurrection of Lamarckism, these recent discoveries on the inheritance of acquired characters must be seen cautiously and should not be used for the rehabilitation of Lysenko, who is considered a fraud [37]. When Serebrovsky proposed his theory, he was labeled as an “enemy of the people” and his work as “pseudoscientific theories in genetics” [38]. Consequently, Serebrovsky’s work was not well-publicized until 1965, long after his death (in 1948) and at the end of Lysenko’s influence on Soviet science.

Although Lysenko’s conclusions on the inheritance of acquired characters could be explained by the studies of several previous researchers, it was based on a literal interpretation of Marx and Engels, who greatly admired Darwin’s theories [37]. However, their theories were published before Mendelian genetics became widely accepted and the inheritance of acquired characters, or Lamarckism, ceased to be considered valid.

## 4. Lesson Learned and Future Threats

The political interference certainly caused a delay in development of science in the USSR and serious problems in agriculture and livestock research [36]. The life and career of T. Lysenko have been extensively analyzed [37]. However, the above-cited evidences should represent a key example for the present times, characterized by frequent and unjustified attacks on science, e.g., from flat-earthers [39], anti-vaccine activists [40], creationists [41], and religious fundamentalists [42]. In this alarming sight, the danger of political and religious interferences on science must be emphasized.

A further key example deals with the coronavirus disease (COVID-19) pandemic, caused by the severe acute respiratory syndrome coronavirus 2 (SARS-CoV-2), which has fostered the repurposing of licensed drugs. In this scenario, high research and public opinion attention has been devoted to the utilization of chloroquine and hydroxychloroquine against SARS-CoV-2 [43]. However, even with tests being currently developed in several countries [44], no sound scientific evidence of the usefulness of their utilization has been found [45,46]. Political reasons, alleging urgency, boosted the use of these drugs. However, like any treatment, especially for such serious and widespread disease, they must not be proposed without sound evidence. Anyway, further research on this issue is required. An even stronger impact of politics on science in these months of COVID-19 pandemics is represented by the COVID-19 pandemic negationism endorsed by governments in several states worldwide [47]. In this pandemics scenario, politics can easily manipulate large masses of people with poor scientific background, with severe risks for public health. A similar situation was experienced—on a smaller scale—few years ago in Italy, when xenophobic political associations pushed the fraudulent theory that migrants imported malaria [48].

To our mind, the present global situation represents a highly worrying scenario. However, there are many people who actively fight to protect science principles and the environment. Broadening our view, Nature’s 10 recently highlighted the admirable position of Ricardo Galvão, working on deforestation in the Amazon and challenging Brazil’s government, as well as the public opinion massive support of the Swedish teenager Greta Thunberg, who combats against the catastrophic impact of climate change, a major contemporary issue widely documented from a scientific point of view [49].

Overall, we believe that science must be defended from political and religious interference by showing the public opinion its methodology and the importance of understanding the world and improving our lives. Anti-science needs to be proved as useless and dangerous, being submitted to careful analysis. In this framework, the role of responsible public opinion leaders in attracting young students toward research-oriented careers has been recently emphasized [49]. Thunberg’s focus on climate change (a topic of high impact also on medical and veterinary entomology, with special reference to the spread of vector-borne diseases [14]) is one of the most beneficial results, which should be considered within the One Health framework. 

One Health needs a delicate and balanced equilibrium to achieve its goals through interdisciplinary collaborations and communications in all aspects of health care for humans, animals, and the environment [50]. The synergism achieved contributes to the acceleration of biomedical research and boosts public health efficacy. The present entomological case study reported here about Lysenko, Serebrovsky, and the screwworm fly control contributes to stress that—among the facets of One Health research at the forefront of biology and medicine—it is crucial to support the development of effective pest and vector management strategies, limiting any political interference.

## 5. Conclusions

Sound scientific evidences are mandatory for the understanding of nature and the management of problems, such as the ones in agricultural and public health. Political and religious considerations must not interfere in these solutions. Lysenko’s interference on scientific issues, such as the development of autocidal control principles, due to coincidence of interest with the USSR leader Stalin, is utilized as an example of such noxious interference. Mostly in our times of climate changes, overpopulation, and pandemics, these interferences must be prevented.
